# MALAT1-miR-101-SOX9 feedback loop modulates the chemo-resistance of lung cancer cell to DDP via Wnt signaling pathway

**DOI:** 10.18632/oncotarget.21693

**Published:** 2017-10-09

**Authors:** Wei Chen, Wei Zhao, Li Zhang, Lixin Wang, Jipeng Wang, Zongren Wan, Yongqing Hong, Liang Yu

**Affiliations:** ^1^ Department of Respiratory Medicine, Huai’an First People’s Hospital, Nanjing Medical University, Huai’an, Jiangsu, China; ^2^ Department of Pathology, Nanjing First Hospital, Nanjing Medical University, Nanjing, Jiangsu, China; ^3^ Department of Hematology, Huai’an First People’s Hospital, Nanjing Medical University, Huai’an, Jiangsu, China

**Keywords:** MALAT1, miR-101, SOX9, lung cancer, chemo-resistance

## Abstract

Cisplatin (DDP)-based chemotherapy is a standard strategy for lung cancer, while chemoresistance remains a major therapeutic challenge. Recent evidence highlights the crucial regulatory roles of long non-coding RNAs (lncRNA) in tumor biology. Metastasis-associated lung adenocarcinoma transcript 1 (MALAT1) has important roles in regulating the proliferation, invasion and migration of lung cancer cell. High MALAT1 expression in lung cancer was related to poorer clinicopathologic features in this study. MALAT1 knockdown alone was sufficient to amplify DDP-induced repression of cell viability. MALAT1 knockdown could also sensitized DDP-resistant lung cancer cells (A549/DDP and H1299/DDP) to DDP. Further assays indicated that MALAT1 acted as a competing endogenous RNA to upregulate SOX9 expression by sponging miR-101 in DDP-resistant cancer cells, through Wnt signaling pathway. Moreover, SOX9 could bind to the promoter of MALAT1 to activate its transcription. Taken together, MALAT1, miR-101 and SOX9 form a feedback loop to enhance the chemo-resistance of lung cancer cell to DDP; this MALAT1-miR-101-SOX9 feedback loop plays an important role in the chemo-resistance of lung cancer cell to DDP and may serve as a potential target for cancer treatment.

## INTRODUCTION

Lung cancer is the most common cause of global cancer-related death. 1.8 million people are diagnosed with lung cancer each year, and 1.6 million people die from the disease. 5-year survival rates range from 4-17% depending on stage and regional differences [1, 2]. DDP is the most common used agent in lung cancer therapy, but the chemo-resistance of lung cancer cells still remains a huge challenge. Therefore, to identify biomarkers that promote early diagnosis and allow personalized therapy for patients, and to figure out the underlying mechanism of the lung cancer cell chemo-resistance has become an urgent need [2, 3].

Human genome sequence data indicate that more than 90% of the DNA sequences are actively transcribed but only 2% encode proteins, thus the majority of transcripts are referred to as non-coding RNAs (ncRNAs) [4, 5]. The roles of small non-coding RNAs such as microRNAs (miRNAs) in gene regulation and cell function have been extensively studied in numerous cancers [5]. MiRNAs have been regarded as essential regulators in resistance to lung cancer treatments [6], including Cisplatin (DDP)-based chemotherapy [7]. In addition to miRNAs, recent studies have shown that long non-coding RNAs (lncRNAs) play an important role in normal development and diseases, including cancer [8]. In addition to cancer cell proliferation, invasion and migration, the roles of lncRNAs in cancer cell chemo-resistance have been frequently reported [9-11].

The mechanisms of lncRNAs affecting drug resistance vary with circumstance; however, the interaction between lncRNAs and microRNAs plays a major role [12, 13]. LncRNA CASC2 interacts with miR-181a to modulate glioma growth and resistance to TMZ through PTEN pathway [9]. LncRNA UCA1 contributes to imatinib resistance by acting as a ceRNA against miR-16 in chronic myeloid leukemia cells [11]. Previously, the important roles of metastasis-associated lung adenocarcinoma transcript 1 (MALAT1) in lung cancer have been well established. MALAT1 is a critical regulator of the metastasis phenotype of lung cancer cells [14], and associated with tumor invasion in non-small cell lung cancer [15]. In addition, MALAT1 is associated with the chemo-resistance of many cancers. MALAT1 can be significantly upregulated by bortezomib and doxorubicin in extramedullary myeloma, thereby acting as a stress response gene and linking chemotherapy to EMM formation [16]. MALAT1 is also associated with poor prognosis to oxaliplatin-based chemotherapy in colorectal cancer patients and promotes chemoresistance through EZH2 [17]. Although the role of MALAT1 was associated with lung cancer progression and prognosis, whether it is involved in the chemo-resistance of lung cancer cell and the underlying mechanism still remains unclear.

In this study, MALAT1, miR-101 and SOX9 formed a feedback loop, which plays a crucial role in regulating the chemo-resistance of lung cancer cell through activation of chemo-resistance-related Wnt signaling pathway. Our findings provide a novel understanding of the role of MALAT1 and this MALAT1-miR-101-SOX9 feedback loop in lung cancer chemo-resistance and the mechanism involved.

## RESULTS

### High MALAT1 expression in lung cancer was related with poorer clinicopathological parameters and shorter overall survival

**Figure 1:**
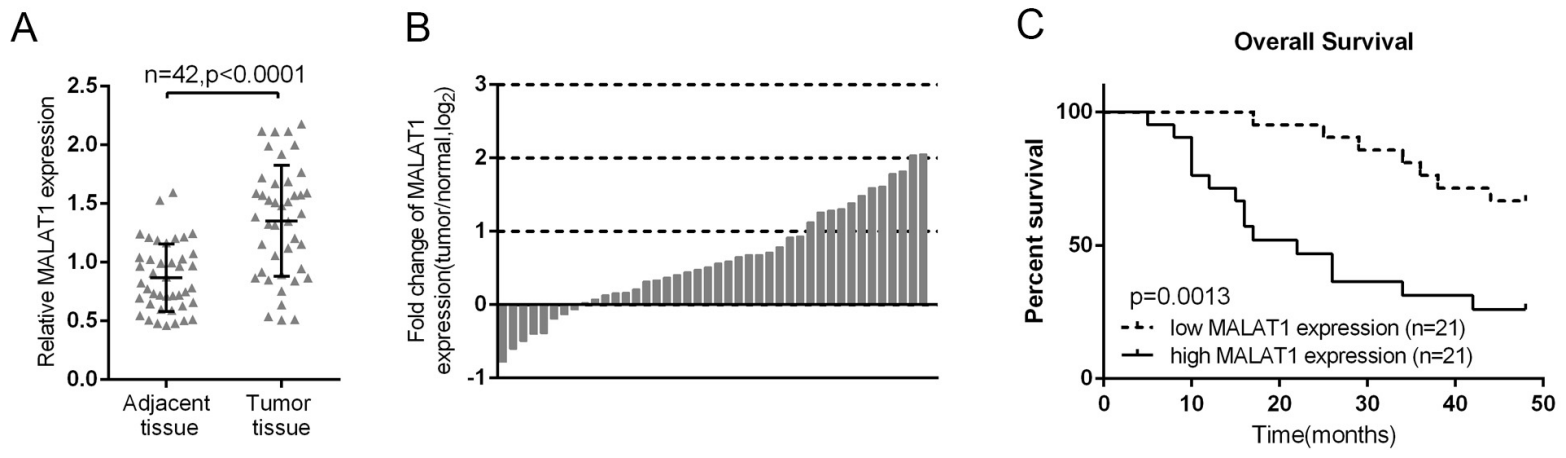
High MALAT1 expression in lung cancer was related with poorer clinicopathological parameters and shorter overall survival **(A)** MALAT1 expression in 42 paired of lung cancer tissues was determined using real-time PCR assays, compared to the adjacent tissues. **(B)** Expression of MALAT1 in 42 paired of lung cancer tissues and their corresponding adjacent non-tumorous tissues (ANTs) in a training cohort. Expression level of MALAT1 was determined by real-time PCR and normalized to U6. Fold change were analyzed using the formula 2-(ΔΔCT [lung cancer/ANT]). **(C)** Kaplan-Meier overall survival curves for 42 patients with lung cancer classified according to relative MALAT1 expression level. The data are presented as mean ± SD of three independent experiments. ^**^P<0.01.

The promotive function of MALAT1 in lung cancer cell viability and proliferation has been well studied [18, 19], and verified here using MTT and BrdU assays (Supplementary Figure 1). The expression of MALAT1 was firstly monitored in lung cancer tissues. In 42 paired lung cancer tissues and the corresponding adjacent tissues, the expression of MALAT1 was significantly increased in lung cancer tissues, compared to the corresponding normal tissues (Figure 1A). To validate this result, quantitative real-time PCR was performed in 42 paired of cases of lung cancer tissues and adjacent normal tissues in training cohort. Compared to the corresponding normal tissues, MALAT1 showed to be significantly up-regulated (more than 2-fold [i.e., log_2_ (fold change) > 1]) in 12 lung cancer cases (> 28.57%) (Figure 1B). 42 paired of cases of lung cancer tissues were divided into two groups: a high MALAT1 expression group (above the median MALAT1 expression, n = 21) and a low MALAT1 expression group (below the median MALAT1 expression, n = 21). High expression of MALAT1 in lung cancer showed to be related with larger tumor size (P=0.031), advanced TNM stage (P=0.013), distant metastasis (P=0.005) and lymph node metastasis (P=0.005) as exhibited in Table 1. To determine the potential relationship between MALAT1 expression and the patients’ prognosis, Kaplan-Meier analysis and log-rank test were used to evaluate the effects of MALAT1 expression on overall survival (OS). The results indicated that patients with higher MALAT1 expression had a significantly poorer prognosis compared to patients with lower MALAT1 expression (P = 0.0013) (Figure 1C). A COX risk proportional regression model was further used to analyze the survival and pathological characteristics of 42 paired of cases. The results of univariate analysis showed that TNM stage, distant metastasis and MALAT1 expression caused significant difference in survival time; the results of multivariate analysis showed that MALAT1 expression caused differences in survival time were statistically significant (P = 0.039, HR = 2.804; 95% CI: 1.053-7.467) (Table 2). However, whether MALAT1 is involved in the chemo-resistance of lung cancer cell still remains unclear.

**Table 1:** Correlation between lncRNA MALAT1 expression and clinicopathological features in lung cancer patients

Parameters	Group	MALAT1 expression		p
		high	low	
Age (years)	<50	13(31.0%)	10(23.8%)	0.352
	≥50	8(19.0%)	11(26.2%)	
Gender	male	15(35.7%)	12(28.6%)	0.334
	female	6(14.3%)	9(21.4%)	
Tumor size (cm)	<3	7(16.7%)	14(33.3%)	0.031
	≥3	14(33.3%)	7(16.7%)	
Histology	Adenoma	10(23.8%)	11(26.2%)	0.758
	Squamous	11(26.2%)	10(23.8%)	
Differentiation	Moderate-Poor	14(33.3%)	13(31.0%)	0.747
	Well	7(16.7%)	8(19.0%)	
TNM stage	I+II	6(14.3%)	14(33.3%)	0.013
	III+IV	15(35.7%)	7(16.7%)	
Lymph node metastasis	Absence	7(16.7%)	16(38.1%)	0.005
	Presence	14(33.3%)	5(11.9%)	
Distant metastasis	Absence	8(19.0%)	17(40.5%)	0.005
	Presence	13(31.0%)	4(9.5%)	

**Table 2:** Univariate and multivariate analysis for factors related to overall survival using the COX proportional hazard model

Characteristics		Univariate analysis			Multivariate analysis		
		p	HR	95%CI	p	HR	95%CI
Age	<50 vs ≥50	0.138	1.975	0.804-4.848	N.A		
Gender	female vs male	0.470	0.718	0.292-1.766	N.A		
Tumor size	<3 vs ≥3	0.194	0.567	0.242-1.333	N.A		
Histology	Adenoma vs Squamous	0.108	0.489	0.205-1.169	N.A		
Differentiation	Moderate-Poor vs Well	0.650	1.231	0.502-3.021	N.A		
TNM stage	I+II vs III+IV	0.008	0.277	0.108-0.715	0.175	0.381	0.094-1.538
Lymph node metastasis	Absence vs Presence	0.431	1.408	0.601-3.299	N.A		
Distant metastasis	Absence vs Presence	0.011	0.327	0.138-0.771	0.847	1.14	0.300-4.325
MALAT1	high vs low	0.003	3.958	1.595-9.825	0.044	2.897	1.031-8.135

### MALAT1 was associated with the chemo-resistance of lung cancer cell

**Figure 2:**
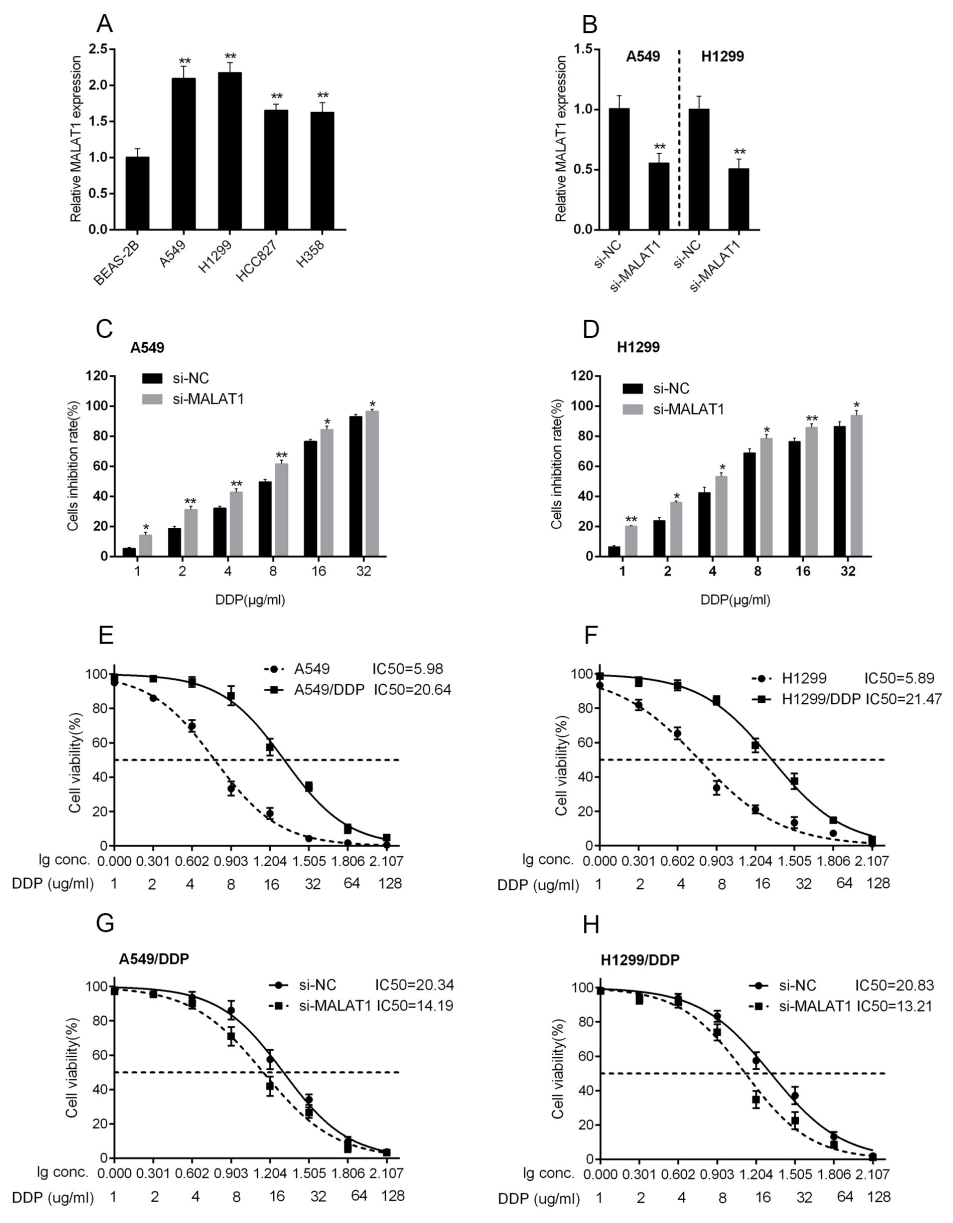
MALAT1 was associated with the chemo-resistance of lung cancer cell **(A)** MALAT1 expression in lung cancer cell lines, A549, H1299, HCC827, H358 and normal cell line, BEAS-2B using real-time PCR assays. **(B)** si-MALAT1 was transfected into A549 and H1299 cells to achieve MALAT1 knockdown, as verified using real-time PCR assays. **(C)** and **(D)** A549 and H1299 cells were exposed to a series of doses of DDP (1, 2, 4, 6, 8, 16, 32 μg/ml); the cell viability of A549 and H1299 cell lines were then determined using MTT assays. The data are presented as mean ± SD of three independent experiments. ^*^P<0.05, ^**^P<0.01. **(E)** and **(F)** A549, DDP-resistant A549 (A549/DDP), H1299, DDP-resistant H1299 (H1299/DDP) cells were treated with a series of doses of DDP (1, 2, 4, 8, 16, 32, 64, 128 μg/ml), and the cell viability of the indicated cells was determined using MTT assays. Data was displayed as a percentage normalized to the viability of cells with no DDP treatment. The abscissa was the logarithm of DDP concentration (log-conc.). LC50 represented the concentration of DDP when cell viability was reduced to 50%. **(G)** and **(H)** A549/DDP and H1299/DDP cells were transfected with siRNA-NC/siRNA-MALAT1. The cell viability was then determined using MTT assays. Data was processed and presented as indicated.

We revealed that high MALAT1 expression is associated with poorer prognosis of lung cancer; here we further investigated whether MALAT1 is associated with the chemo-resistance of lung cancer cell. The expression of MALAT1 were at a significantly higher level in four human lung cancer cell lines, A549, H1299, HCC827 and H358, compared to normal cell, BEAS-2B (Figure 2A). Among the four cell lines, MALAT1 expressed at higher levels in A549 and H1299 cell lines. A si-MALAT1 vector was transfected into A549 and H1299 cell lines to achieve MALAT1 knockdown, as verified using real-time PCR assays (Figure 2B). Then these si-MALAT1-transfected A549 and H1299 cells were exposed to DDP treatment (1, 2, 4, 6, 8, 16, 32 μg/ml) to validate the inhibitory effect of DDP on lung cancer cell viability. Results showed that DDP could significantly inhibit the viability of both A549 and H1299 cells in a dose-dependent manner (Figure 2C and 2D). Then A549, A549/DDP, H1299 and H1299/DDP cells were treated with a series dose of DDP (1, 2, 4, 8, 16, 32, 64, 128 μg/ml), and then the cell viability was monitored. The cell viability of cells with no treatment was defined as 100%. Results showed that for A549 cells, the DDP concentration to reduce cell viability to 50% was about 5.98 μg/ml (lC50 = 5.98); for A549/DDP cells this value was 20.64 μg/ml (lC50 = 20.64) (Figure 2E). Similar results were observed for H1299 cells, the DDP concentration to reduce H1299 cell viability to 50% was about 5.89 μg/ml (lC50 = 5.89), for H1299/DDP cells 21.47 μg/ml (lC50 = 21.47) (Figure 2F). A549/DDP and H1299/DDP cells were transfected with si-NC or si-MALAT1, and then repeated the above assays to validate the effect of MALAT1 on lung cancer cells’ chemo-sensitivity. Results showed that MALAT1 knockdown significantly amplified DDP-induced repression of lung cancer cells viability, and reduced the lC50 values from 20.34 (A549/DDP) and 20.83 (H1299/DDP) to 14.19 (A549/DDP) and 13.21 (H1299/DDP) (Figure 2G and 2H). These data suggested that MALAT1 could induce the lung cancer cell chemo-resistance to DDP-based chemo-therapy. However, the mechanism by which MALAT1 regulates the chemo-resistance of lung cancer cells to DDP remains to be investigated.

### The interaction of MALAT1, miR-101 and SOX9

**Figure 3:**
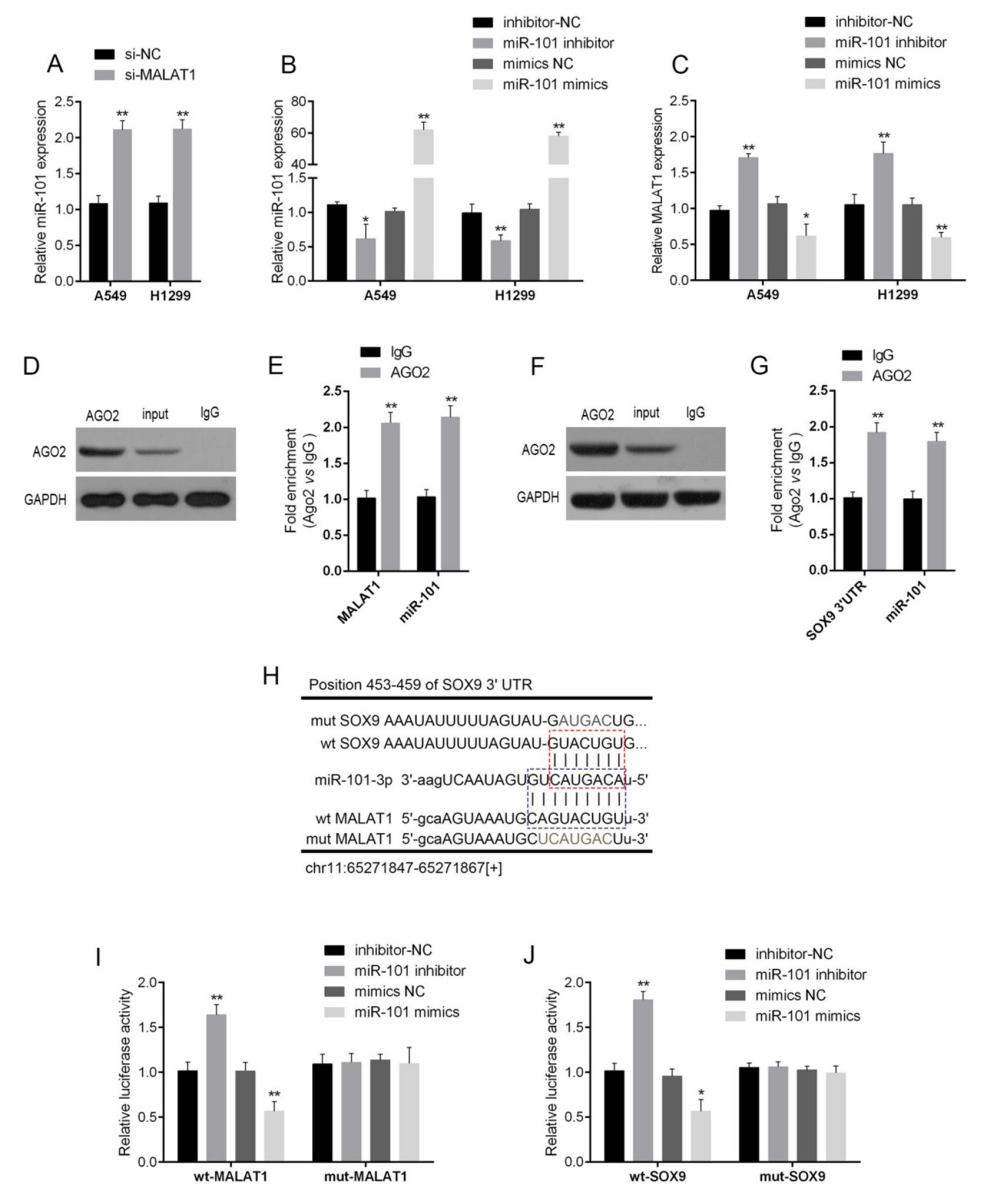
The interaction of MALAT1, miR-101 and SOX9 **(A)** si-MALAT1 was transfected into A549 and H1299 to achieve MALAT1 knockdown; the expression of miR-101 in A549 and H1299 cells was determined using real-time PCR assays. **(B)** MiR-101 mimics or miR-101 inhibitor was transfected into A549 and H1299 cells to achieve ectopic miR-101 expression or miR-101 inhibition, as verified using real-time PCR assays, and **(C)** the expression of MALAT1 in A549 and H1299 cells was determined using real-time PCR assays. **(D)** and **(E)** Association of miR-101 and MALAT1 with AGO2. A549 cellular lysates were used for RNA immunoprecipitation with AGO2 antibody. Detection of AGO2 and IgG using Western blot (up), and detection of miR-101 or MALAT1 using qRT-PCR (low). All data of MALAT1 expression were normalized to β-actin mRNA expression levels. MiR-101 expression data was normalized to U6 small RNA expression. **(F)** and **(G)** Association of SOX9 and MALAT1 with AGO2. **(H)** A wild-type and mutated MALAT1 (wt-MALAT1 and mut-MALAT1 containing a 9 bp mutation in the predicted binding sites of miR-101) or SOX9 3’UTR (wt-SOX9 3’UTR and mut-SOX9 3’UTR containing a 7 bp mutation in the predicted binding sites of miR-101) luciferase reporter gene vector was constructed. **(I)** and **(J)** The indicated vectors were co-transfected into A549 cells with miR-101 mimics or miR-101 inhibitor, and the luciferase activity was then determined using the Dual Luciferase assays. The data are presented as mean ± SD of three independent experiments. ^*^P<0.05, ^**^P<0.01.

LncRNAs interact with miRNAs to exert their functions. Herein, the candidate miRNAs of MALAT1 were predicted using StarBase, and 17 of the candidates were associated with chemo-resistance. Among them, miR-101 was the most strongly promoted in A549 and H1299 cells by MALAT1 knockdown (Supplementary Figure 2, Supplementary Tables 1 and 2). As verified using real-time PCR, miR-101 expression was significantly up-regulated after si-MALAT1 transfection in A549 and H1299 cells (Figure 3A). Next, miR-101 mimics or miR-101 inhibitor was transfected into lung cancer cells to achieve ectopic miR-101 expression or miR-101 inhibition, verified using real-time PCR assays (Figure 3B). MALAT1 expression in response to ectopic miR-101 expression or miR-101 inhibition was then determined using real-time PCR assays. Results showed that MALAT1 could be significantly down-regulated by ectopic miR-101 expression, whereas up-regulated by miR-101 inhibition (Figure 3C). These data suggested the potential interaction between MALAT1 and miR-101.

Given that miR-101 inhibits MALAT1 expression, suggesting that there might be the RNA-induced silencing complex (RISC complex) in both miR-101 and MALAT1. Previously, SOX9 has been regarded as the direct target of miR-101. In several cancers, miR-101 inhibits SOX9 by direct targeting to suppress cancer cell proliferation, migration and invasion [20, 21], suggesting that there might be RISC complex in both miR-101 and SOX9. Argonaute2 (Ago2) promotes the target mRNA degradation or inhibit its protein translation; it has been regarded as the core component of RISC [22]. Here, we further investigated the interaction between MALAT1 and miR-101, miR-101 and SOX9 in lung cancer cells using RNA immunoprecipitation assays with the AGO2 antibody. As exhibited by Western blot assays, AGO2 protein could be precipitated from the cellular extract (Figure 3D and 3F). In RNA extracted from the precipitated AGO2 protein, both miR-101 and MALAT1 obtained a more than 2-folds enrichment compared to IgG (Figure 3E), both miR-101 and SOX9 with a 1.8∼2-folds enrichment compared to IgG (Figure 3G), indicating that miR-101, MALAT1 and SOX9 existed in RISC.

To further validate these two interactions (MALAT1/miR-101, miR-101/SOX9), Luciferase assays were employed by construction of a wt/mut-MALAT1 and a wt/mut-SOX9 3’UTR luciferase reporter gene vector (Figure 3H). These indicated vectors were co-transfected into A549 cells with miR-101 mimics or miR-101 inhibitor; then the luciferase activity was determined using dual luciferase assays. Results showed that the luciferase of wt-MALAT1 and wt-SOX9 3’UTR vectors was suppressed by miR-101 mimics, amplified by miR-101 inhibitor; after the mutation in the predicted miR-101 binding site in MALAT1 or the 3’UTR of SOX9, the changes of the luciferase was abolished (Figure 3I and 3J), indicating that MALAT1 could directly bind to miR-101, and miR-101 could directly bind to the 3’UTR of SOX9.

### Wnt signaling was involved in the acquisition of the chemo-resistance of lung cancer cell

**Figure 4:**
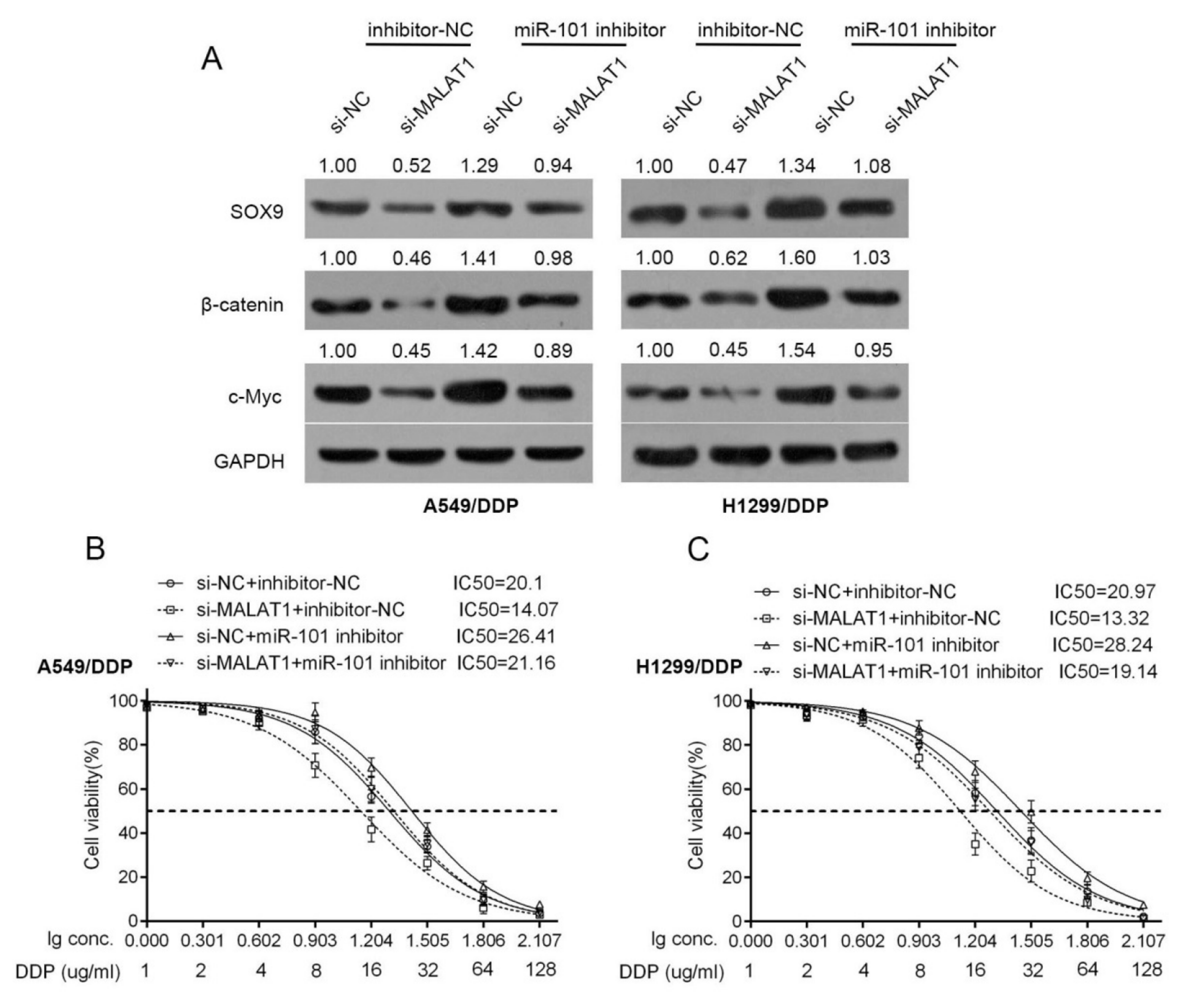
Wnt signaling was involved in the acquisition of the chemo-resistance of lung cancer cell **(A)** A549/DDP and H1299/DDP cells were co-transfected with si-MALAT1 and miR-101 inhibitor; the protein levels of SOX9, β-catenin and c-myc in A549/DDP and H1299/DDP cells were determined using Western blot assays. **(B)** and **(C)** A549/DDP and H1299/DDP cells were co-transfected with si-MALAT1 and miR-101 inhibitor; the cell viability was then determined using MTT assays. Data was processed and presented as indicated.

β-catenin is a crucial component of Wnt signaling, which is involved in developmental processes, tumorigenesis and the chemo-resistance of cancer cell [23-25, 26]. Here, we investigated whether β-catenin and its downstream target gene, c-myc, is involved in the chemo-resistance of lung cancer cell. A549/DDP and H1299/DDP cells were co-transfected with miR-101 inhibitor and si-MALAT1; then the protein levels of SOX9, β-catenin and c-myc were determined using Western blot assays. Results showed that MALAT1 knockdown significantly reduced the protein levels of SOX9, β-catenin and c-myc; miR-101 inhibition significantly increased the protein levels of SOX9, β-catenin and c-myc; the effect of MALAT1 knockdown on the indicated proteins could be partially reversed by miR-101 inhibition (Figure 4A).

Further, the effects of co-processing si-MALAT1 and miR-101 inhibitor on the chemo-resistance of lung cancer cell were evaluated. Results showed that MALAT1 knockdown sensitize the lung cancer cell to DDP (brought down the lC50 value of A549/DDP cell from 20.1 to 14.07, of H1299/DDP cell from 20.97 to 13.32); miR-101 inhibition promoted the chemo-resistance of the lung cancer cell to DDP (promoted the lC50 value of A549/DDP cell to 26.41, of H1299/DDP cell to 28.24); moreover, the effect of MALAT1 knockdown on the chemo-resistance of the lung cancer cell could be partially reversed by miR-101 inhibition (reversed the lC50 value of A549/DDP cell from 14.07 to 21.16, of H1299/DDP cell from 13.32 to 19.14) (Figure 4B and 4C).

### SOX9 could bind to the promoter of MALAT1 to promote MALAT1 expression

**Figure 5:**
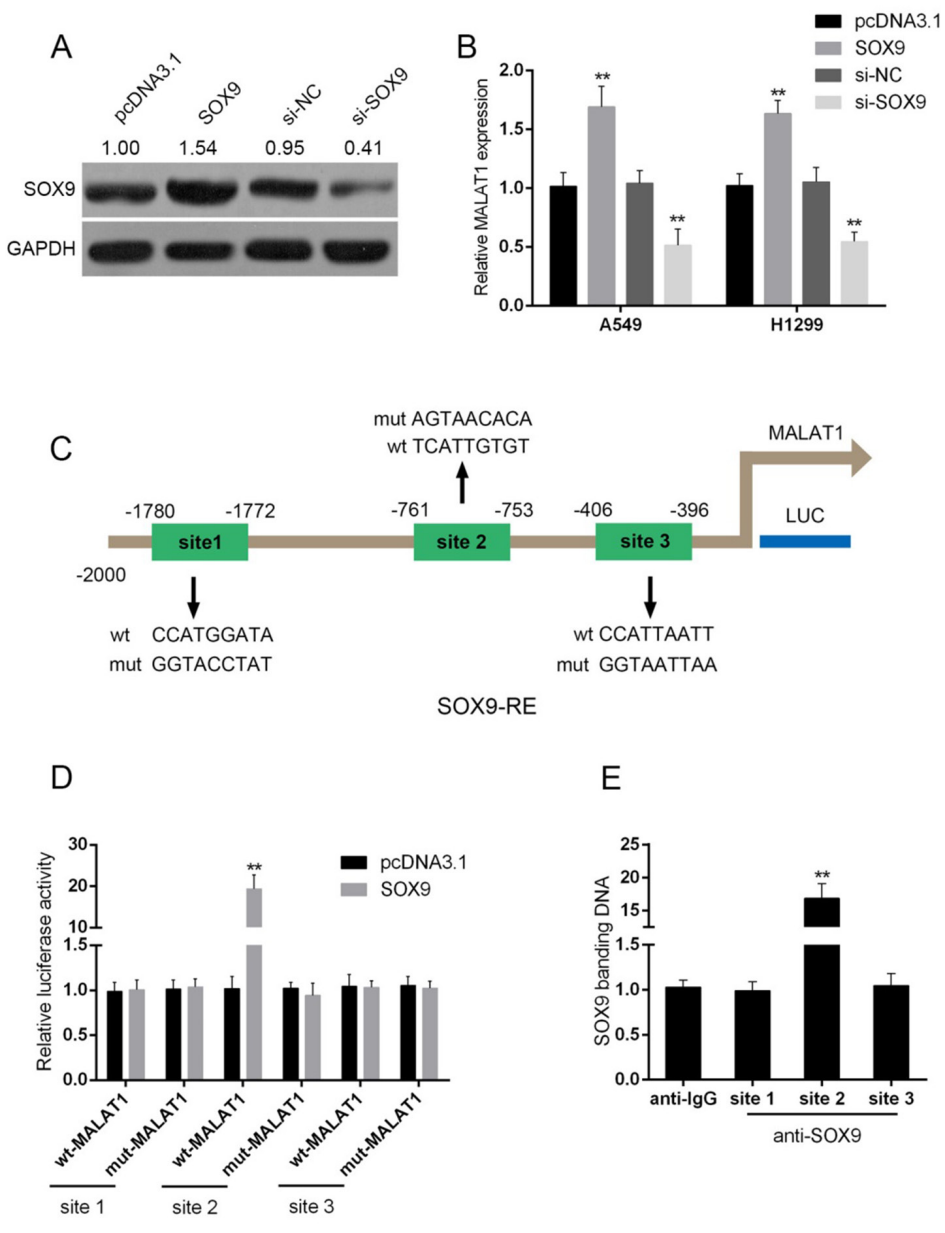
SOX9 could bind to the promoter of MALAT1 to promote MALAT1 expression **(A)** pcDNA3.1/SOX9 or si-SOX9 was transfected into A549 and H1299 cells to achieve forced SOX9 expression or SOX9 knockdown, as verified using Western blot assays. **(B)** MALAT1 expression in response to SOX9 overexpression or SOX9 knockdown was determined using real-time PCR assays. **(C)** A schematic diagram of potential SOX9 binding element (three possible binding sites) in the promoter region of the MALAT1 gene predicted by Jaspar database. A wt-MALAT1 promoter luciferase reporter vector and a mut-MALAT1 promoter luciferase reporter vector were constructed. **(D)** The indicated vectors were co-transfected into A549 cells with pcDNA3.1/SOX9; the luciferase activity was determined. **(E)** The real-time ChIP assay showed that the level of SOX9 antibody binding to MALAT1 promoter was much greater than that of IgG. The data are presented as mean ± SD of three independent experiments. ^**^P<0.01.

To investigate the detailed role and the possible mechanism of MALAT1 and SOX9 interaction, pcDNA3.1/SOX9 was transfected into A549 and H1299 to achieve forced SOX9 expression, as verified using Western blot assays (Figure 5A). In A549 and H1299 cells, MALAT1 was up-regulated by forced SOX9 expression, down-regulated by anti-SOX9-induced SOX9 knockdown (Figure 5B). Jaspar database predicted that MALAT1 promoter possesses SOX9-reactive-element (SOX9RE, Figure 5C). Through mutating three predicted binding sites, a wt-MALAT1 (without mutation) and a mut-MALAT1 (containing a 9 bp mutation in any of the three predicted binding sites) luciferase reporter gene vector were constructed (Figure 5C). These indicated vectors were co-transfected into A549 cells, and then the luciferase activity was determined. Results showed that the SOX9 significantly amplified the luciferase activity as compared to pcDNA3.1 when co-transfected with wt-MALAT1 containing site 2. When binding element was mutated on site 2, luciferase activity was not changed, compared to that of the pcDNA3.1 (Figure 5D). Furthermore, the real-time ChIP assay showed that the level of SOX9 antibody binding to site 2 of the binding elements in the MALAT1 promoter was much greater than that of IgG (Figure 5E), suggesting that SOX9 might bind to the promoter of MALAT1 on the predicted site 2 to activate its expression.

### A schematic diagram showing the mechanism by which MALAT1/miR-101/SOX9 affects the chemo-resistance of lung cancer cell

**Figure 6:**
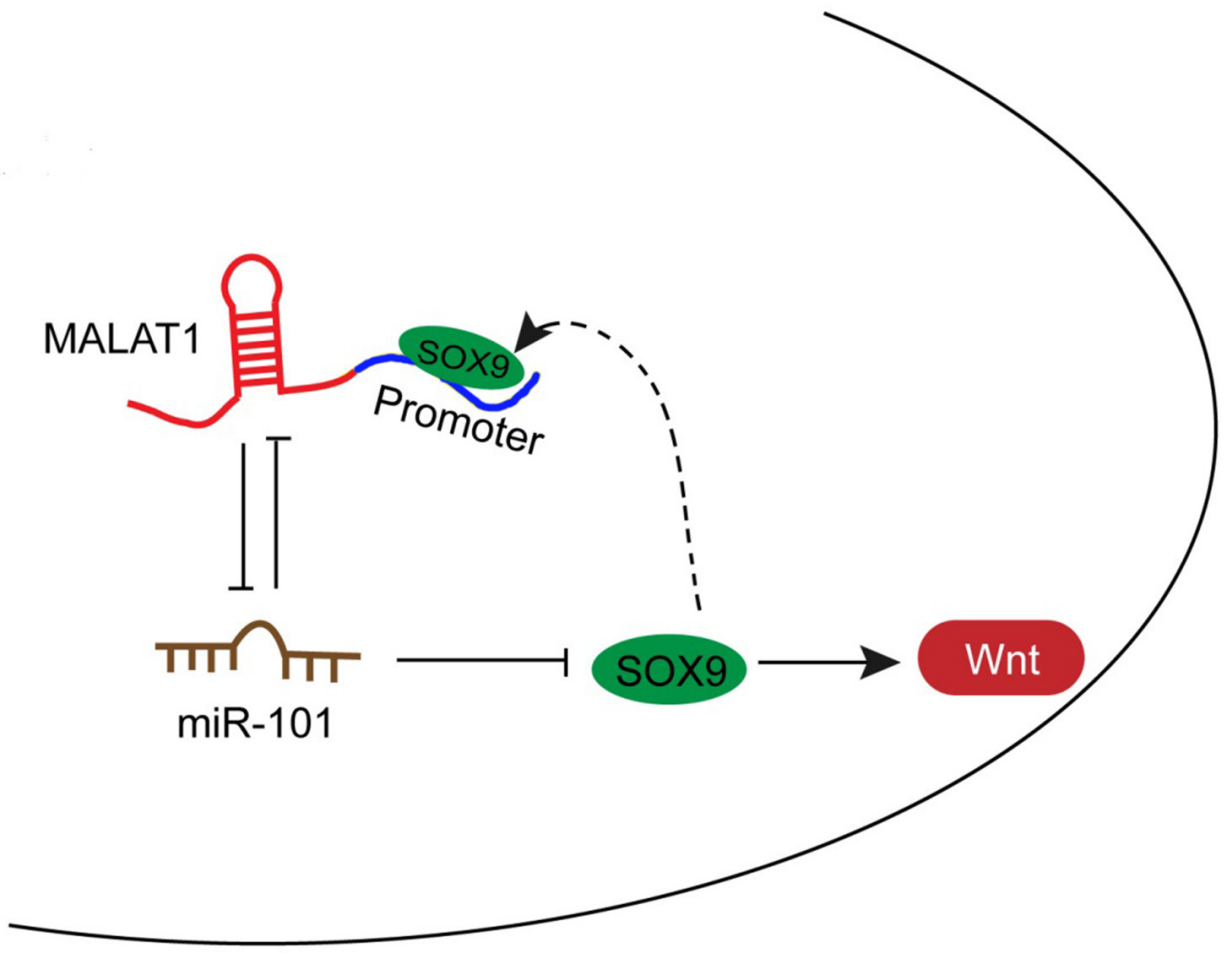
A schematic diagram showing the mechanism by which MALAT1/miR-101/SOX9 affects the chemo-resistance of lung cancer cell

In the present study, we revealed that MALAT1 inhibits miR-101 expression by direct targeting; moreover, MALAT1 competes with SOX9 for miR-101 binding, so as to inhibit miR-101 expression and promote SOX9 expression. Further, through promoting the protein levels of Wnt signaling-related factors, the chemo-resistance of lung cancer cell to DDP is enhanced (Figure 6).

On the other hand, the promoter of MALAT1 possesses a SOX9RE (three possible sites), which enable SOX9 to bind to the promoter of MALAT1 to activate its expression. Subsequently, enhanced MALAT1 expression promotes SOX9 expression, so as to enhance the chemo-resistance of lung cancer cell to DDP (Figure 6).

## DISCUSSION

The non-coding portion of the genome accounts for more than 90% of the total mammalian genome. Studies have demonstrated that genetic variations and epigenetic modification, such as miRNA and lncRNA regulation, have emerged as a novel research field in DNA repair pathways which influence chemotherapeutic outcome [27]. The roles of dysregulated lncRNAs and miRNAs in the chemo-resistance of many kinds of cancers, including lung cancer, have garnered increased scientific interest in recent years [27].

Accumulating evidence confirms that lncRNAs can affect the sensitivity of cancer cells to chemo-therapy. HOTAIR contributes to chemo-resistance of lung adenocarcinoma and cervical cancer via inhibiting p21 expression [28, 29]. Another lncRNA, UCA1, enhances 5-fluorouracil resistance in colorectal cancer by inhibiting miR-204-5p [30]. MALAT1 is associated with poor response to oxaliplatin-based chemotherapy in colorectal cancer patients and promotes chemoresistance through EZH2 [31]. In the present study, we confirmed a high expression of MALAT1, a well-studied oncogenic lncRNA in lung cancer, is associated with poorer clinicopathologic features, which has inspired us to further investigate the potential role of MALAT1 in chemo-resistance of lung cancer cells. We observed that MALAT1 knockdown could amplify the suppressive effect of DDP on the cell viability of A549 and H1299 cells. Subsequent cell viability assays performed on DDP-resistant lung cancer cells (A549/DDP and H1299/DDP) showed that MALAT1 knockdown reduced the lC50 values for both A549/DDP and H1299/DDP cells, indicating MALAT1 knockdown sensitizes DDP-resistant lung cancer cells to DDP.

However, the mechanism by which MALAT1 affects the chemo-sensitivity of lung cancer cells remains to be investigated. It has recently been discovered that the interactions between lncRNAs and miRNAs influence post-transcriptional regulation by inhibiting available miRNA activity. Moreover, miRNAs act on their downstream genes to inhibit their expression. According to previous studies, miR-101 plays an essential role in cancers, including lung cancer. After miR-101 being sponged by SPRY4-IT1, the proliferation and metastasis of bladder cancer can be promoted [32]. Through blocking PI3K/AKT pathway, miR-101-3p inhibits the growth and metastasis of non-small cell lung cancer [33]. More importantly, miR-101 overexpression enhances the cytotoxic effect of anti-cancer drugs through inhibiting the proliferation of colon cancer cell [34]. In the present study, we demonstrated the interaction between MALAT1 and miR-101, between miR-101 and SOX9, a direct downstream target of miR-101 [20, 21], in lung cancer cells through RNA immunoprecipitation and Luciferase assays. Interestingly, MALAT1 shared almost the same miR-101 binding site with SOX9, suggesting that MALAT1 may compete with SOX9 for miR-101 binding, thereby inhibiting miR-101 expression and promoting SOX9 expression. Luan et al. reported that MALAT1 acts as a competing endogenous RNA to promote malignant melanoma growth and metastasis by sponging miR-22 [35]. MALAT1 functions as a competing endogenous RNA to regulate MCL-1 expression by sponging miR-363-3p in gallbladder cancer [36]. In the present study, a consistent result was observed: the SOX9 protein was reduced after MALAT1 knockdown, whereas promoted after miR-101 inhibition; the effect of MALAT1 knockdown on SOX9 protein could be partially reversed by miR-101 inhibition. Here, we revealed that MALAT1 might act as a competing endogenous RNA to promote SOX9 expression through inhibiting miR-101 for the first time.

Previously, SOX9 has been reported to activate Wnt signaling to promote cancer progression and the chemo-resistance of cancer cell [37-40]. Here, we also investigated whether the Wnt signaling is involved in the chemo-resistance of lung cancer cell. As verified by Western blot assays, the protein levels of β-catenin and c-myc was significantly reduced by MALAT1 knockdown, promoted by miR-101 inhibition, same as SOX9 protein. This indicates that Wnt signaling is involved in the chemo-resistance of lung cancer cell. Since MALAT1 knockdown could amplify the suppressive effect of DDP on lung cancer cell viability; we further investigated the detailed function of MALAT1/miR-101 in regulating the chemo-resistance of lung cancer cell. Consistent with our earlier results, MALAT1 knockdown reduced the lC50 values of lung cancer cells; moreover, miR-101 inhibition significantly promoted the lC50 values, and partially reversed the effect of MALAT1 on lung cancer cell chemo-resistance.

Interestingly, in addition to the interaction with miR-101, we also observed a positive regulation of MALAT1 by SOX9. What is the underlying mechanism of SOX9 promoting MALAT1 expression? Jaspar database predicts that the promoter of MALAT1 possesses a SOX9RE. Through Luciferase and real-time ChIP assays, we confirmed that SOX9 could bind to the promoter of MALAT1 on the predicted site 2 to activate its expression. We already revealed that MALAT1 competes with SOX9 for miR-101, as well as miR-101 directly binds to SOX9 to inhibit SOX9 expression; this SOX9RE in the promoter of MALAT1 suggested that MALAT1, miR-101 and SOX9 forms a feedback loop to activate Wnt signaling pathway, finally acts on the chemo-resistance of lung cancer cell.

In summary, we demonstrated that MALAT1 plays an important role in the chemo-resistance of lung cancer by functioning as a ceRNA to regulate the expression of SOX9 by inhibiting miR-101 through the downstream Wnt signaling. In addition to the interaction with miR-101, SOX9 binds to the promoter of MALAT1 to promote MALAT1 expression, thereby enhancing the chemo-resistance of lung cancer. We demonstrated the MALAT1-miR-101-SOX9 feedback loop in lung cancer for the first time; this MALAT1-miR-101-SOX9 feedback loop may potentially act as an effective therapeutic candidate combined with traditional DDP-based chemo-therapy for lung cancer.

## MATERIALS AND METHODS

### Tissue samples, cell lines and reagents

42 paired primary lung cancer tissues and the matched adjacent normal tissues were collected from patients who underwent surgical resection at Huai’an First People’s Hospital, Nanjing Medical University (Huai’an, China) from April 2011 to March 2012. The tissues were snap-frozen in liquid nitrogen. This project was approved by the Ethic Committee of Huai’an First People’s Hospital, Nanjing Medical University. Follow-up and survival time were analyzed. The follow-up periods arrange from 1 month to 48 months.

Immortalized human bronchial epithelial cell, BEAS-2B, human lung cancer cell lines, A549, H1299, HCC827 and H358, and DDP-resistant lung cancer cell line, A549/DDP and H1299/DDP were purchased from the American Type Culture Collection (Manassas, VA, USA). Cells were cultured in RPMI-1640 medium (Invitrogen, Carlsbad, CA, USA) supplemented with 10% fetal bovine serum (Gibco, CA, USA) at 37°C in a humidified atmosphere with 5% CO_2_.

The expression of miR-101 was achieved by transfection of miR-101 mimics or miR-101 inhibitor (Genepharma, Shanghai, China) using Lipofectamine 2000 (Invitrogen). A si-MALAT1 (GeneCopoecia, Guangzhou, China) was used to achieve MALAT1 knockdown. Primers for si-MALAT1 were forward, 5’- TCTAGATTTTTCTTAACAGCT-3’ and reverse, 5’-CTGTTAAGAAAAATCTAGAAA-3’. A pcDNA3.1/SOX9 was used to achieve the overexpression of SOX9, and an anti-SOX9 was used to achieve the knockdown of SOX9 (GeneCopoecia, Guangzhou, China). Cells were plated in 6-well plates or 96-well plates, transfected, incubated for 24 h or 48 h and used for further assays or RNA/protein extraction.

### RNA extraction and SYBR green quantitative PCR analysis

Total RNA was extracted from cells using Trizol reagent (Invitrogen, CA, USA) and detected mature miR-101 expressions in cells using a Hairpin-it TM miRNAs qPCR kit (Genepharma, Shanghai, China). Primers for miR-101 were forward, 5’-GGCGTACAGTACTGTGATA-3’. Expression of RNU6B was used as an endogenous control. Primers for RNU6B were forward, 5’-CTCGCTTCGGCAGCACA-3’ and reverse, 5’-AACGCTTCACGAATTTGCGT-3’. MALAT1 expression was measured by SYBR green qPCR assay (Takara, Dalian, China). Primers for MALAT1 were forward, 5’- AAGAAGCCGAAATAAATGAG-3’ and reverse, 5’-AGCCCACAGGAACAAGTC- 3’. Data were processed using 2^-ΔΔCT^ method.

### MTT assay

Cell viability was evaluated by modified MTT assay. After cultured for 24 h in 96-well plates (5000 cells per well), cells were transfected with si-MALAT1 or co-transfected with si-MALAT1 and miR-101 inhibitor. Medium with DDP (1, 2, 4, 6, 8, 16, 32, 64, 128 μg/ml) was applied at 24 h post-transfection. 48 h after transfection, 20 μl MTT (at a concentration of 5 mg/ml; Sigma-Aldrich) was added, and the cells were incubated for an additional 4 h in a humidified incubator. 200 μl DMSO was added after the supernatant discarded to dissolve the formazan. OD_490 nm_ value was measured. The viability of the non-treatment cells (control) was defined as 100%, and the viability of cells from all other groups was calculated separately from that of the control group.

### BrdU incorporation assay

DNA synthesis in proliferating cells was determined by measuring 5-Bromo-2-deoxyUridine (BrdU) incorporation. BrdU assays were performed at 24 h and 48 h after transfecting A549 and H1299 cells with si-NC or si-MALAT1. After seeding the infected cells in 96-well culture plates at a density of 2 × 10^3^ cells/well, they were cultured for 24 h or 48 h, and incubated with a final concentration of 10 μM BrdU (BD Pharmingen, San Diego, CA, USA) for 2 h to 24 h. When the incubation period ended, the medium was removed, A549 and H1299 cells were fixed for 30 min at RT and incubated with peroxidase-coupled anti-BrdU-antibody (Sigma-Aldrich) for 60 min at RT, washed three times with PBS, incubated with peroxidase substrate (tetramethylbenzidine) for 30 min. The absorbance values were measured at 450 nm. Background BrdU immunofluorescence was determined in cells not exposed to BrdU but stained with the BrdU antibody.

### Western blot analysis

The expression of SOX9, β-catenin and c-Myc in lung cancer cells was detected by performing immunoblotting. Cultured or transfected cells were lysed in RIPA buffer with 1% PMSF, and loaded protein onto a SDS-PAGE minigel and transferred them onto PVDF membrane, and probed with the following antibodies: SOX9 (1:1000, ab185966, Abcam, MA, USA), β-catenin (1:1000, ab32572, Abcam), c-Myc (1:10000, ab32072, Abcam) and GAPDH (1:1000, ab8245, Abcam) at 4°C overnight, the blots were subsequently incubated with HRP-conjugated secondary antibody (1:5000). ECL Substrates was used to visualize signals (Millipore, MA, USA).

### RNA immunoprecipitation

RNA immunoprecipitation assays were performed by using the Imprint RNA Immunoprecipitation Kit (Sigma, St. Louis, USA) along with the AGO2 antibody (Cell signaling, Rockford, USA). The AGO2 antibody was then recovered by protein A/G beads. MALAT1, miR-101 and SOX9 RNA levels in the immunoprecipitates were measured by qRT-PCR.

### Chromatin immunoprecipitation (ChIP)

ChIP was done according to our previous process [36]. Briefly, the treated cells were cross-linked with 1% formaldehyde, sheared by sonication to an average size of 400 bp DNA, and immunoprecipitated using antibodies against SOX9 (anti-SOX9, AMAB90795, CL0639, monoclonal, 1:1000, Sigma-Aldrich). A positive control antibody (RNA polymerase II) and a negative control non-immune IgG were used to demonstrate the efficacy of the kit reagents (Epigentek Group Inc., NY, USA, P-2025-48). The immunoprecipitated DNA was subsequently cleaned, released, and eluted. The eluted DNA was used for downstream applications, such as ChIP-PCR. The fold-enrichment (FE) was calculated as the ratio of the amplification efficiency of the ChIP sample to that of the non-immune IgG. The amplification efficiency of RNA Polymerase II was used as a positive control. FE% = 2 (IgG CT-Sample CT) × 100%.

### Luciferase reporter assay

A549 cells were seeded into a 24-well plate. Wild-type and mutated MALAT1 (wt-MALAT1 and mut-MALAT1 containing a 9 bp mutation in the predicted binding sites of miR-101) or SOX9 3’UTR (wt-SOX9 3’UTR and mut-SOX9 3’UTR containing a 7 bp mutation in the predicted binding sites of miR-101) or MALAT1 promoter (wt-MALAT1 and mut-MALAT1 containing a 9 bp mutation in three predicted sites of SOX9 responsive element, SOX9RE) luciferase reporter gene vector was constructed. After cultured overnight, cells were co-transfected with the indicated vectors and miR-101 mimics and miR-101 inhibitor, respectively. Luciferase assays were performed 48 h after transfection using the Dual Luciferase Reporter Assay System (Promega, WI, USA).

### Statistical analysis

Data were exhibited as mean ± SD of three independent experiments and processed using SPSS 17.0 statistical software (SPSS, Chicago, IL, USA). P values of <0.05 were considered statistically significant.

## SUPPLEMENTARY MATERIALS FIGURES AND TABLES


