# Supplementary material for: MALAT1-miR-101-SOX9 feedback loop modulates the chemo-resistance of lung cancer cell to DDP via Wnt signaling pathway
Source: Oncotarget. 2017 Oct 9;8(55):94317–29. doi: 10.18632/oncotarget.21693 (PMC5706876; doi:10.18632/oncotarget.21693)
Supplement: Supplementary file 1 [file oncotarget-08-94317-s001.pdf]

MALAT1-miR-101-SOX9 feedback loop modulates the chemo-resistance of lung cancer cell to DDP via Wnt signaling pathway

SUPPLEMENTARY MATERIALS

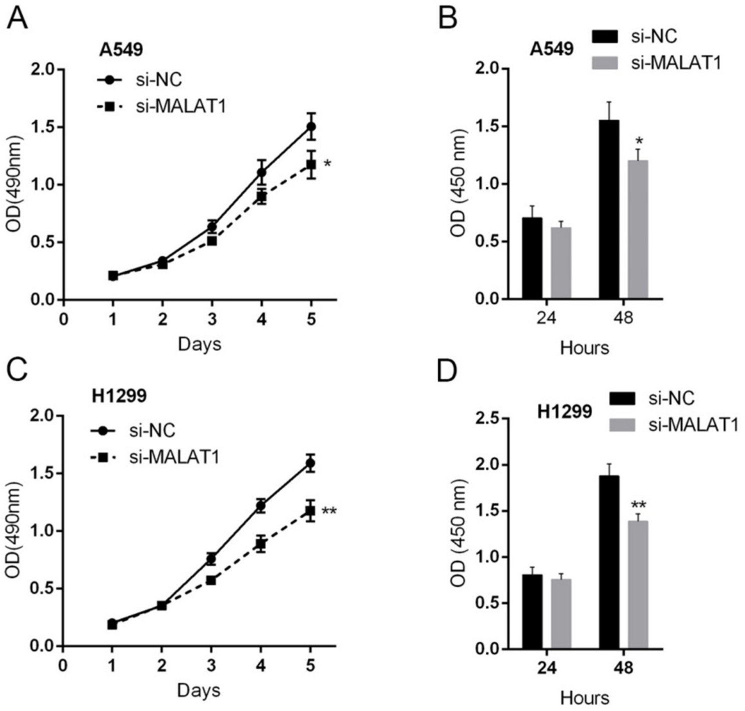

Supplementary Figure 1: The function of MALAT1 knockdown in regulation of lung cancer cell. A549 and H1299 cells were transfected with si-MALAT1. The cell viability and proliferation was determined using MTT and BrdU assays. **(A, C)** The cell viability of A549 and H1299 cells in response to MALAT1 knockdown determined by MTT assays. **(B, D)** The DNA synthesis ability of A549 and H1299 cells in response to MALAT1 knockdown determined by BrdU assays. The data are presented as mean ± SD of three independent experiments. *P<0.05, **P<0.01.

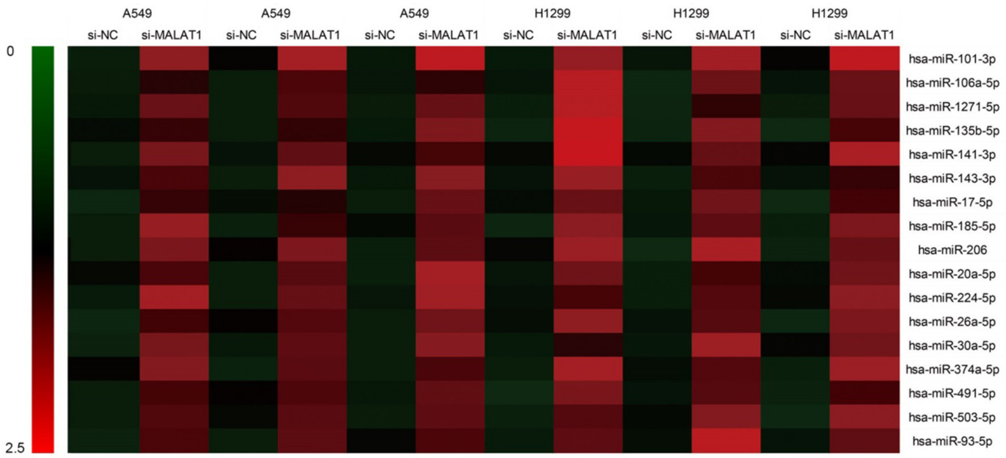

Supplementary Figure 2: The candidate miRNAs of MALAT1 scanned using Starbase. There were 113 miRNAs associated with MALAT1, 17 of them were associated with DDP resistance (NCBI). MALAT1 was down-regulated in A549 and h1299, and the expression of the candidate miRNAs was up-regulated, among which miR-101 expression was the most strongly up-regulated and stable.

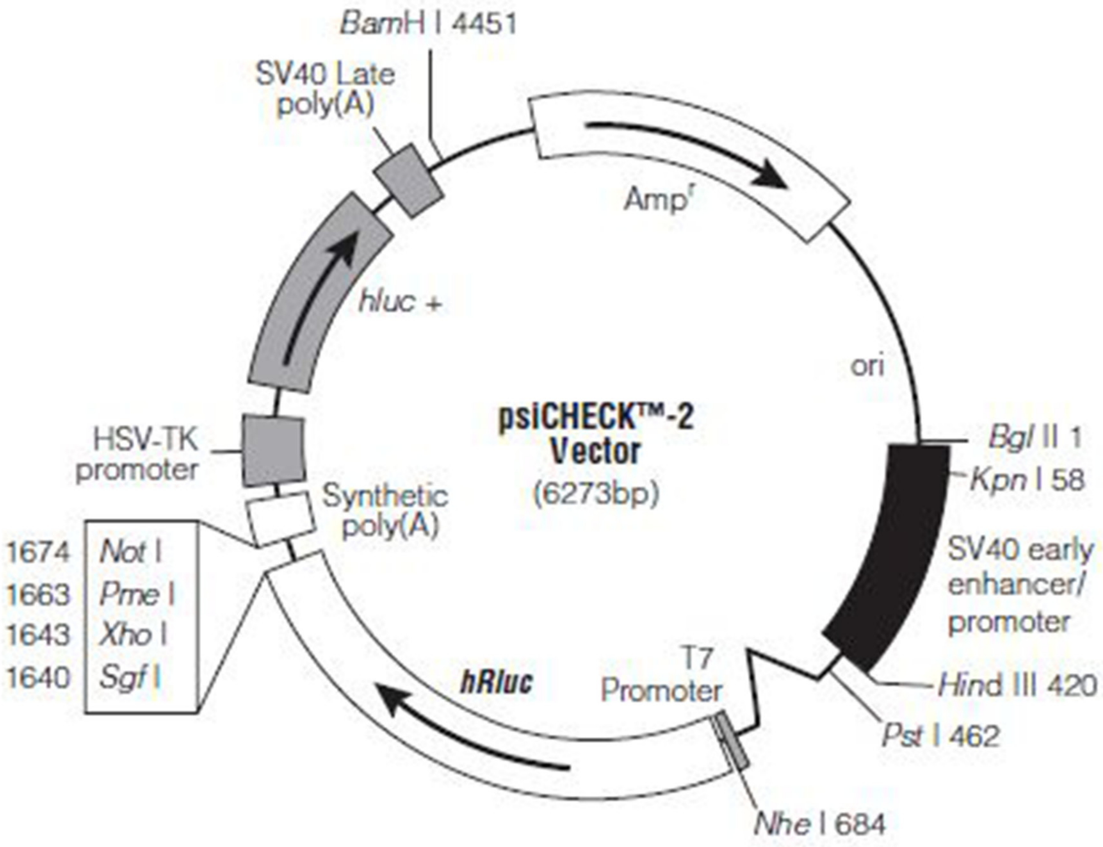

Supplementary Figure 3: The structure of the luciferase reporter gene vector. The structure of psiCHECK™-2 vector used in the present study was shown here. The wild-type of mutant-type 3'UTR of SOX9 or MALAT1 fragment was cloned into the downstream of the Renilla psiCHECK2 vector shown here.

Supplementary Table 1 : Changes of miRNA expression in response to MALAT1 knockdown in A549 cells

A549	si-NC					si-MALAT1					
	a1	a2	a3	average 1	sd	a1	a2	a3	average 2	sd	average 2/ average 1
hsa-miR-101-3p	1.000	1.207	1.046	1.084	0.109	1.986	2.107	2.237	2.110	0.126	1.946
hsa-miR-106a-5p	1.015	1.000	1.065	1.027	0.034	1.443	1.636	1.476	1.519	0.103	1.479
hsa-miR-1271-5p	1.050	1.000	1.010	1.020	0.027	1.788	1.659	1.765	1.737	0.069	1.703
hsa-miR-135b-5p	1.147	1.000	1.033	1.060	0.077	1.523	1.497	1.895	1.639	0.223	1.546
hsa-miR-141-3p	1.000	1.112	1.172	1.095	0.087	1.858	1.735	1.601	1.731	0.128	1.582
hsa-miR-143-3p	1.111	1.000	1.054	1.055	0.055	1.622	1.982	1.953	1.852	0.200	1.756
hsa-miR-17-5p	0.890	1.135	1.000	1.009	0.123	1.518	1.431	1.771	1.573	0.177	1.560
hsa-miR-185-5p	1.000	0.964	1.157	1.040	0.102	2.030	1.525	1.701	1.752	0.256	1.684
hsa-miR-206	1.000	1.228	0.985	1.071	0.136	1.880	1.884	1.717	1.827	0.095	1.706
hsa-miR-20a-5p	1.167	1.000	0.968	1.045	0.107	1.625	1.690	2.104	1.807	0.260	1.729
hsa-miR-224-5p	1.019	1.000	1.072	1.030	0.038	2.096	1.757	2.081	1.978	0.192	1.920
hsa-miR-26a-5p	0.896	1.224	1.000	1.040	0.167	1.574	1.671	1.821	1.689	0.125	1.624
hsa-miR-30a-5p	0.946	1.039	1.000	0.995	0.047	1.864	1.721	1.944	1.843	0.113	1.853
hsa-miR-374a-5p	1.209	0.947	1.000	1.052	0.138	1.925	1.703	1.627	1.751	0.155	1.665
hsa-miR-491-5p	1.000	1.221	1.045	1.089	0.117	1.595	1.644	1.745	1.661	0.076	1.526
hsa-miR-503-5p	1.000	1.164	1.010	1.058	0.092	1.654	1.698	1.728	1.693	0.037	1.600
hsa-miR-93-5p	0.941	1.000	1.178	1.040	0.123	1.631	1.719	1.635	1.662	0.050	1.598

The values shown in Supplementary Table 1 are the relative expression of candidate miRNAs after MALAT1 knockdown in A549 cells. a1, a2 and a3 are relative expression level from each independent experiment; sd stands for standard deviation. MiR-101-3p and miR-224-5p were the most strongly upregulated by MALAT1 knockdown.

Supplementary Table 2: Changes of miRNA expression in response to MALAT1 knockdown in H1299 cells

H1299	si-NC					si-MALAT1					
	a1	a2	a3	average 1	sd	a1	a2	a3	average 2	sd	average 2/ average 1
hsa-miR-101-3p	1.031	1.081	1.191	1.101	0.082	2.012	2.091	2.260	2.121	0.127	1.927
hsa-miR-106a-5p	1.085	0.897	1.100	1.027	0.113	2.209	1.809	1.789	1.936	0.237	1.884
hsa-miR-1271-5p	0.996	0.905	1.012	0.971	0.058	2.196	1.489	1.779	1.821	0.355	1.876
hsa-miR-135b-5p	0.921	0.917	0.868	0.902	0.029	2.286	1.924	1.606	1.939	0.340	2.150
hsa-miR-141-3p	1.163	1.160	1.187	1.170	0.015	2.299	1.756	2.139	2.065	0.279	1.765
hsa-miR-143-3p	1.108	0.970	1.106	1.062	0.079	2.041	1.630	1.519	1.730	0.275	1.630
hsa-miR-17-5p	1.147	1.041	0.864	1.018	0.143	1.778	1.826	1.588	1.731	0.126	1.701
hsa-miR-185-5p	0.908	1.063	0.988	0.986	0.078	1.973	1.724	1.885	1.861	0.126	1.886
hsa-miR-206	1.172	0.864	0.943	0.993	0.160	2.057	2.135	1.769	1.987	0.193	2.001
hsa-miR-20a-5p	1.090	0.995	1.152	1.079	0.079	1.815	1.595	1.823	1.744	0.129	1.617
hsa-miR-224-5p	1.114	0.987	1.167	1.089	0.093	1.605	1.670	1.978	1.751	0.199	1.607
hsa-miR-26a-5p	1.124	1.104	0.894	1.041	0.127	1.990	1.683	1.886	1.853	0.156	1.780
hsa-miR-30a-5p	1.031	1.060	1.185	1.092	0.082	1.459	2.069	1.836	1.788	0.308	1.637
hsa-miR-374a-5p	1.000	1.137	0.969	1.035	0.090	2.101	1.668	2.063	1.944	0.240	1.878
hsa-miR-491-5p	0.865	1.100	0.945	0.970	0.120	1.868	1.685	1.579	1.711	0.146	1.764
hsa-miR-503-5p	0.964	1.161	0.881	1.002	0.144	1.673	1.927	1.973	1.857	0.161	1.854
hsa-miR-93-5p	1.017	1.132	1.100	1.083	0.059	1.726	2.219	1.732	1.892	0.283	1.747

The values shown in Supplementary Table 2 are the relative expression of candidate miRNAs after MALAT1 knockdown in H1299 cells. a1, a2 and a3 are relative expression level from each independent experiment; sd stands for standard deviation. MiR-101-3p, miR-135b-5p and miR-206 were the most strongly upregulated by MALAT1 knockdown.
